# Use of Artificial Intelligence–Generated Synthetic Data to Augment and Enhance the Performance of Clinical Prediction Models in Patients With Alcohol-Associated Hepatitis and Acute Cholangitis

**DOI:** 10.1016/j.gastha.2025.100643

**Published:** 2025-02-22

**Authors:** Joseph C. Ahn, Yung-Kyun Noh, Mingzhao Hu, Xiaotong Shen, Vijay H. Shah, Patrick S. Kamath

**Affiliations:** 1 Division of Gastroenterology and Hepatology, Mayo Clinic, Rochester, Minnesota; 2 Department of Computer Science, Hanyang University, Seoul, South Korea; 3 Division of Clinical Trials and Biostatistics, Department of Quantitative Health Sciences, Mayo Clinic, Rochester, Minnesota; 4 School of Statistics, University of Minnesota, Minneapolis, Minnesota

Artificial intelligence (AI) and machine learning (ML) algorithms hold immense promise for enhancing clinical prediction and improving outcomes in the field of gastroenterology and hepatology.^1^ However, the performance of complex ML algorithms is heavily dependent on the size and quality of the training dataset.^2^ In many scenarios, obtaining large high-quality datasets from diverse populations can be challenging due to factors such as patient privacy concerns, data sharing restrictions, and the inherent rarity of conditions.^3^ This scarcity of real-world data can significantly hamper the development and optimization of ML models, potentially limiting their clinical utility.

Synthetic data present an innovative solution to this challenge. Defined as “data that have been generated using a purpose-built mathematical model or algorithm, with the aim of solving a (set of) data science task(s),”^4^ synthetic data can augment existing datasets or even replace real data entirely in certain scenarios. In health care, synthetic medical datasets show promise in advancing research and innovation while addressing key challenges in data access and privacy.^5^ Among the methods for generating synthetic data, diffusion models have emerged as state-of-the-art approach. Diffusion models are a class of generative AI techniques that work by gradually adding noise to data and then learning to reverse this process, ultimately generating new high-quality samples.^6^ Diffusion models offer advantages over algorithms such as generative adversarial networks and variational autoencoders in data quality and coherence, training stability, ability to handle high-dimensional data, sample diversity, and ability to preserve privacy of the original data.^6^^,^^7^ Diffusion models have demonstrated remarkable success in generating high-quality synthetic data across various domains.^8^ While widely adopted for image generation in creative and technical fields, these models are equally capable of generating structured, tabular data such as electronic health records.^9^ In this study, we perform a proof-of-concept investigation using diffusion models to generate a synthetic cohort based on our previously published dataset of alcohol-associated hepatitis (AH) and acute cholangitis (AC) patients.^10^ We hypothesize that ML algorithms trained on this larger synthetic dataset will match or exceed the performance of those trained on the original data when tested on an external real-world cohort.

Our original study utilized 2 real-world cohorts for ML model development and external validation.^10^ The primary cohort, used for model development and internal validation, consisted of 459 adult patients admitted to Mayo Clinic, Rochester, between January 1, 2010, and December 31, 2019. This cohort included 265 patients with AH and 194 patients with AC due to endoscopic retrograde cholangiopancreatography–confirmed choledocholithiasis. Patients were identified using International Classification of Diseases (ICD) Ninth Revision and ICD Tenth Revision codes, with diagnoses confirmed by manual chart review. For external validation, we utilized data from the Medical Information Mart for Intensive Care III database, comprising 305 adult patients admitted to Beth Israel Deaconess Medical Center in Boston between 2001 and 2012. This external cohort included 92 admissions for AH and 213 admissions for AC, identified using ICD Ninth Revision codes. For both cohorts, 10 laboratory variables were collected as input features for the ML algorithms: white blood cell count, hemoglobin, mean corpuscular volume, platelet count, aspartate aminotransferase, alanine aminotransferase, alkaline phosphatase, total bilirubin, direct bilirubin, and albumin.

We developed a diffusion model to generate synthetic data for AH and AC patients based on a denoising diffusion probabilistic model framework (Supplementary Material). We visually compared the distribution of synthetic data to the original data using scatter plots of key laboratory parameters. The synthetic dataset showed remarkable similarity to the original dataset in kernel density estimation plot (Figure). A box plot representation also showed that variables in the synthetic dataset had very similar median values and interquartile ranges as those in the original dataset (Figure A1). The maximum mean discrepancy for the 2 datasets was close to 0 around 0.04, indicating that the 2 distributions are nearly identical (Figure A2).

**Figure:**
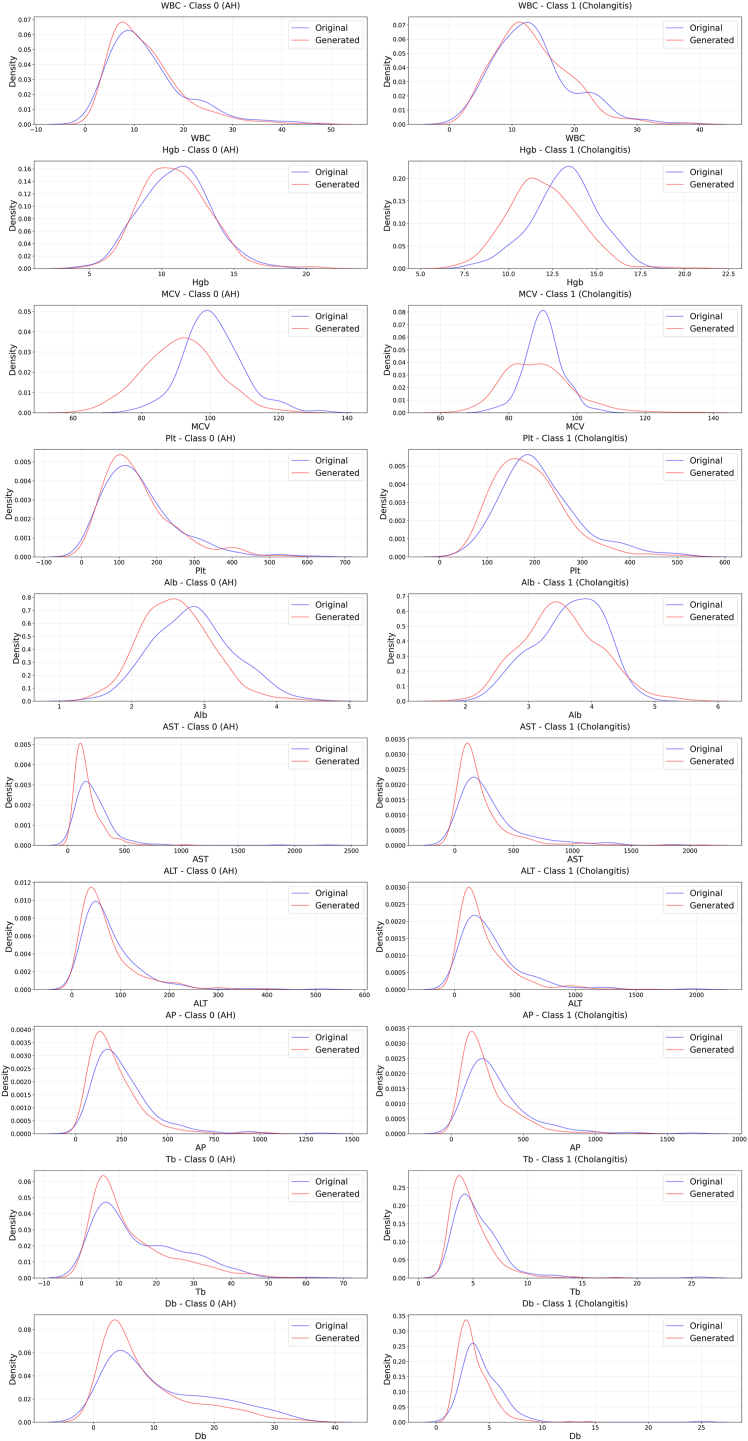
Comparison of real and synthetic data distributions for laboratory parameters in AH and AC patients. The figure shows kernel density estimation plots comparing the distribution of real (blue) and synthetic (red) data for 10 laboratory parameters across 2 patient classes (0 and 1). Class 0 represents AH patients, while Class 1 represents AC patients. The overlapping distributions demonstrate the high fidelity of the synthetic data in replicating the statistical properties of the real data across all parameters and both patient classes. Alb, albumin; ALT, alanine aminotransferase; AP, alkaline phosphatase; AST, aspartate aminotransferase; Db, direct bilirubin; Hgb, hemoglobin; MCV, mean corpuscular volume; Plt, platelet count; Tb, total bilirubin; WBC, white blood cell count.

To evaluate the utility of the synthetic data, we trained 7 different ML classification algorithms for classifying AH vs AC using the 10 laboratory values. We then tested their performance on the real-world external validation set from the Medical Information Mart for Intensive Care III database. Strikingly, the ML algorithms trained using synthetic data consistently outperformed those trained using the original Mayo data, with higher accuracies across all algorithms (k-nearest neighbors: 0.894 vs 0.865, logistic regression: 0.900 vs 0.882, support vector machine: 0.935 vs 0.865, decision tree: 0.882 vs 0.824, Naive Bayes: 0.800 vs 0.659, artificial neural network: 0.924 vs 0.882, and random forest: 0.841 vs 0.782, *P* < .01) (Table A1).

These results highlight the feasibility and potential of generated synthetic data to not only replicate key features of real-world clinical data but also enhance the performance of prediction models. The implications of this study extend beyond the immediate results, offering transformative possibilities for clinical research. Diffusion model–generated synthetic data can foster a new collaborative research paradigm by facilitating secure data sharing among institutions. This approach also offers a solution for data augmentation, addressing the persistent issues of minority underrepresentation and class imbalance in medical datasets. Moreover, synthetic control arms, which simulate comparison groups in clinical trials, offer a promising way to accelerate the development of new treatments. This approach is particularly valuable in rare diseases or in situations where traditional randomized trials are logistically difficult or costly.

Several limitations should be considered in the context of our findings. Synthetic data may not capture rare but clinically significant edge cases and could potentially amplify existing biases in training data. While our models showed promising results, synthetic data might only represent patterns from the original dataset without capturing full real-world variability. Study-specific limitations include: (1) synthetic data generation based on single-center data may not fully capture patient diversity across health-care settings; (2) focus on 10 laboratory parameters excludes other potentially important clinical features; (3) evaluation limited to binary classification rather than more complex clinical scenarios; (4) analysis of single time point data doesn’t capture disease progression patterns; and (5) fixed model architecture without extensive evaluation of alternatives. There are also risks of overfitting to synthetic data artifacts, though our external validation helps mitigate this concern. Privacy and regulatory frameworks for synthetic data validation also need development. Future research should focus on expanding to higher-dimensional clinical scenarios with diverse populations, establishing standardized validation protocols including bias assessment, developing techniques to capture clinically relevant edge cases, optimizing real-world data integration, and developing regulatory guidelines for implementation. By addressing these challenges while leveraging synthetic data's potential, we can work toward more robust clinical prediction models in gastroenterology and hepatology.
