# Supplementary material for: Use of Artificial Intelligence–Generated Synthetic Data to Augment and Enhance the Performance of Clinical Prediction Models in Patients With Alcohol-Associated Hepatitis and Acute Cholangitis
Source: Gastro Hep Adv. 2025 Feb 22;4(6):100643. doi: 10.1016/j.gastha.2025.100643 (PMC12138892; doi:10.1016/j.gastha.2025.100643)
Supplement: Supplementary Material [file mmc1.docx]

**Supplementary Material:**

**Understanding Diffusion Models - A Simplified Explanation**

Diffusion models can be understood through a simple analogy of blurring and deblurring an image:

1. Forward Process (Adding Noise):

- Start with real patient data (like a clear photograph)
- Gradually add random noise in small steps (like slowly adding fog)
- Eventually, the data becomes pure noise (completely foggy)

1. Learning Process:

- The model learns how this noise was added
- More importantly, it learns how to reverse this process

1. Generation Process (Removing Noise):

- Start with random noise (like a foggy picture)
- Gradually remove noise in small steps
- End up with new, synthetic data that looks realistic

This process ensures:

- Generated data maintains important patterns from real data
- Each generated case is unique (not copied from real patients)
- Patient privacy is protected since we start from random noise
- The model captures complex relationships between variables

In our study, we apply this technique to laboratory values, allowing us to generate synthetic patient data that preserves the clinical relationships found in real patient records while maintaining privacy.

**Detailed Methodology for Diffusion Model and Synthetic Data Generation**

**1. Data Preparation**

Our study utilized a dataset of 459 patients from Mayo Clinic, Rochester, comprising 265 cases of alcohol-associated hepatitis (AH) and 194 cases of acute cholangitis (AC). We selected ten key laboratory variables as input features: white blood cell count, hemoglobin, mean corpuscular volume, platelet count, albumin, aspartate aminotransferase, alanine aminotransferase, alkaline phosphatase, total bilirubin, and direct bilirubin.

To address the common issue of skewness in medical data, we applied a log transformation to all features. This step helps in normalizing the data distribution and can improve the performance of machine learning models.

**2. Diffusion Model Architecture**

We developed a denoising diffusion probabilistic model (DDPM) using the PyTorch framework. The core of our model is a neural network designed to estimate noise, which we call the "noise prediction network." This network consists of six hidden layers, each containing 2000 neurons, allowing for complex feature representation and noise estimation.

To incorporate time information into our model, we implemented sinusoidal position embeddings for time steps. This technique, inspired by transformers in natural language processing, allows the model to understand and utilize the temporal aspect of the diffusion process.

For handling class information (AH or AC), we employed conditional embeddings. These embeddings allow the model to generate class-specific samples and enable controlled generation of synthetic data.

**3. Diffusion Process**

Our diffusion process follows a linear noise schedule over 1000 steps. The noise level, represented by β, ranges from a minimum of 10^-4 to a maximum of 0.02. This gradual increase in noise allows for a smooth transition from the original data distribution to pure noise.

The forward process involves progressively adding noise to the original data points. Conversely, the backward process, which is learned by our model, aims to reverse this noising process, step by step removing noise to generate synthetic data points.

**4. Model Training**

We trained our model for 1000 epochs using the Adam optimizer, with a learning rate of 0.001. The training process involves a technique called classifier-free guidance. In this approach, we randomly drop class information during training, which enables more controlled generation of synthetic data later on.

For each training step, we:

1. Sample a batch of real data points.
2. Generate random noise.
3. Sample a random time step.
4. Apply noise to the real data according to the sampled time step.
5. Use our model to predict the added noise.
6. Calculate the loss between the predicted and actual noise.
7. Update the model parameters to minimize this loss.

This process allows the model to learn how to reverse the noising process effectively.

**5. Synthetic Data Generation**

After training, we generate synthetic data using a process that reverses the forward diffusion:

1. We start with pure Gaussian noise.
2. Over 1000 steps, we gradually denoise this initial noise using our trained model.
3. At each step, we apply classifier-free guidance. This involves generating two predictions: one with class information and one without. We then combine these predictions using a weighting parameter 'w'.
4. The weighting parameter 'w' controls the strength of class conditioning. A higher 'w' results in synthetic data more strongly conforming to class characteristics.

**Complete Source Code for Diffusion Model**

import torch

import torch.nn as nn

import torch.nn.functional as F

import math

class SinusoidalPositionEmbedding(nn.Module):

"""Time embedding using sinusoidal positions"""

def __init__(self, dim):

super().__init__()

self.dim = dim

def forward(self, time):

device = time.device

half_dim = self.dim // 2

embeddings = math.log(10000) / (half_dim - 1)

embeddings = torch.exp(torch.arange(half_dim, device=device) * -embeddings)

embeddings = time[:, None] * embeddings[None, :]

embeddings = torch.cat((embeddings.sin(), embeddings.cos()), dim=-1)

return embeddings

class EmbeddingBlock(nn.Module):

"""Neural network block for embedding conditioning information"""

def __init__(self, input_dim, emb_dim):

super().__init__()

self.input_dim = input_dim

self.model = nn.Sequential(

nn.Linear(input_dim, emb_dim),

nn.GELU(),

nn.Linear(emb_dim, emb_dim),

nn.Unflatten(1, (emb_dim,))

)

def forward(self, x):

x = x.view(-1, self.input_dim)

return self.model(x)

class NoisePredictionNetwork(nn.Module):

"""Core network for predicting noise in the diffusion process"""

def __init__(self, input_dim, output_dim, total_steps, hidden_dims=[2000]*6,

time_embed_dim=8, class_embed_dim=2):

super().__init__()

self.total_steps = total_steps

# Main layers

self.layers = nn.ModuleList([

nn.Linear(input_dim, hidden_dims[0]),

*[nn.Linear(hidden_dims[i], hidden_dims[i+1])

for i in range(len(hidden_dims)-1)],

nn.Linear(hidden_dims[-1], output_dim)

])

# Embeddings

self.time_embed = SinusoidalPositionEmbedding(time_embed_dim)

self.time_proj = nn.ModuleList([

EmbeddingBlock(time_embed_dim, dim)

for dim in hidden_dims[:2]

])

self.class_proj = nn.ModuleList([

EmbeddingBlock(class_embed_dim, dim)

for dim in hidden_dims[:2]

])

def forward(self, x, t, class_labels, class_mask):

# Time embedding

t = t.float() / self.total_steps

t = self.time_embed(t)

t_emb = [proj(t) for proj in self.time_proj]

# Class conditioning

c = class_labels * class_mask

c_emb = [proj(c) for proj in self.class_proj]

# Forward pass through layers with conditioning

h = torch.relu(self.layers[0](x))

h = torch.relu(self.layers[1](c_emb[0]*h + t_emb[0]))

h = torch.relu(self.layers[2](c_emb[1]*h + t_emb[1]))

for layer in self.layers[3:]:

h = torch.relu(layer(h))

return h

class DDPM(nn.Module):

"""Denoising Diffusion Probabilistic Model"""

def __init__(self, network, n_steps=1000, min_beta=1e-4, max_beta=0.02, device=None):

super().__init__()

self.n_steps = n_steps

self.device = device

self.network = network.to(device)

# Setup noise schedule

self.betas = torch.linspace(min_beta, max_beta, n_steps).to(device)

self.alphas = 1 - self.betas

self.alpha_bars = torch.tensor([torch.prod(self.alphas[:i + 1])

for i in range(len(self.alphas))]).to(device)

def forward(self, x0, t, noise=None):

"""Forward diffusion process"""

n, d = x0.shape

a_bar = self.alpha_bars[t]

if noise is None:

noise = torch.randn(n, d).to(self.device)

# Add noise according to diffusion schedule

noisy = a_bar.sqrt().reshape(n, 1) * x0 + (1 - a_bar).sqrt().reshape(n, 1) * noise

return noisy

def backward(self, x, t, class_labels, class_mask):

"""Reverse diffusion process with classifier-free guidance"""

return self.network(x, t, class_labels, class_mask)


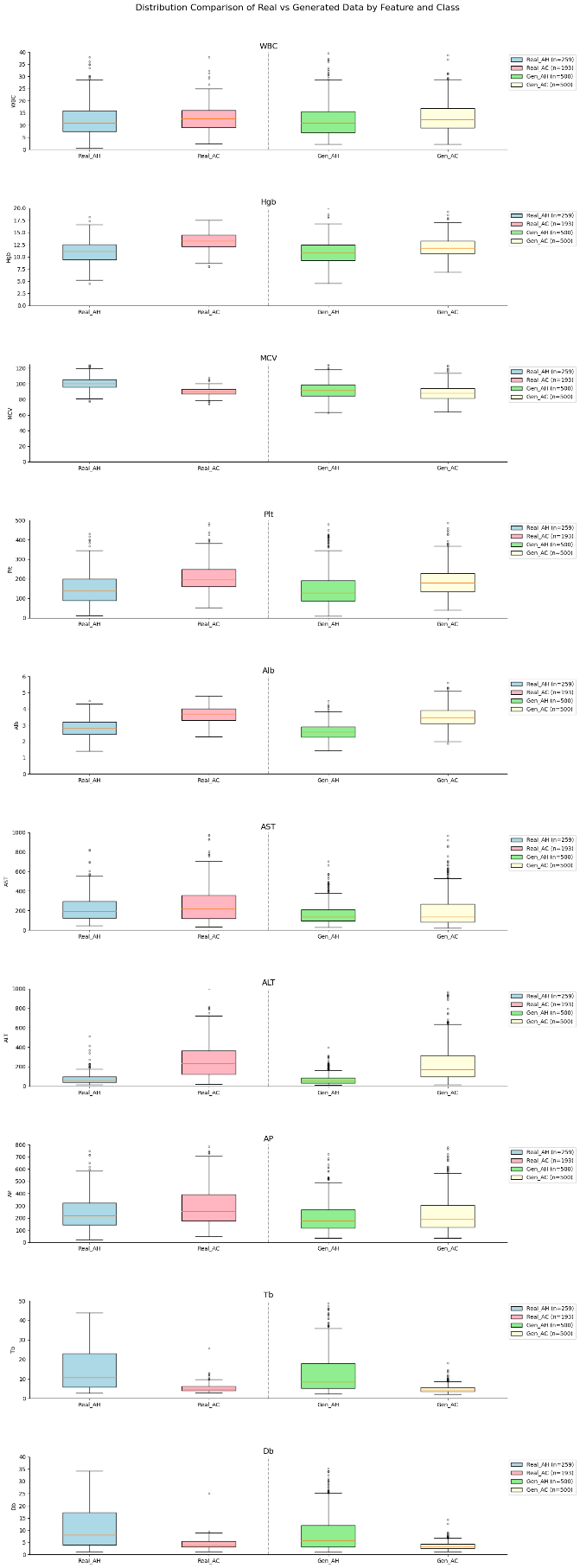
**Supplementary Figure 1: Box Plot Comparison of Real and Synthetic Data Distributions for Laboratory Parameters in Alcohol-associated Hepatitis and Acute Cholangitis Patients**

The figure presents box plots comparing the distribution of real and synthetic data for ten laboratory parameters in Alcohol-associated Hepatitis (AH) and Acute Cholangitis (AC) patients.

Color coding: Blue: Real AH data Red: Real AC data Green: Synthetic AH data Yellow: Synthetic AC data

Parameters shown: WBC: White Blood Cell count (x10^3/μL) Hgb: Hemoglobin (g/dL) MCV: Mean Corpuscular Volume (fL) Plt: Platelet count (x10^3/μL) Alb: Albumin (g/dL) AST: Aspartate Aminotransferase (U/L) ALT: Alanine Aminotransferase (U/L) AP: Alkaline Phosphatase (U/L) Tb: Total Bilirubin (mg/dL) Db: Direct Bilirubin (mg/dL)

Each box plot displays the median (central line), interquartile range (box), and outliers (points) for each parameter. The similar distributions between real and synthetic data for both AH and AC groups demonstrate the high fidelity of the synthetic data in replicating the statistical properties of the real data across all parameters.

**Supplementary Figure 2: Maximum Mean Discrepancy (MMD) Scores Between Real and Synthetic Data Across Laboratory Parameters**

**
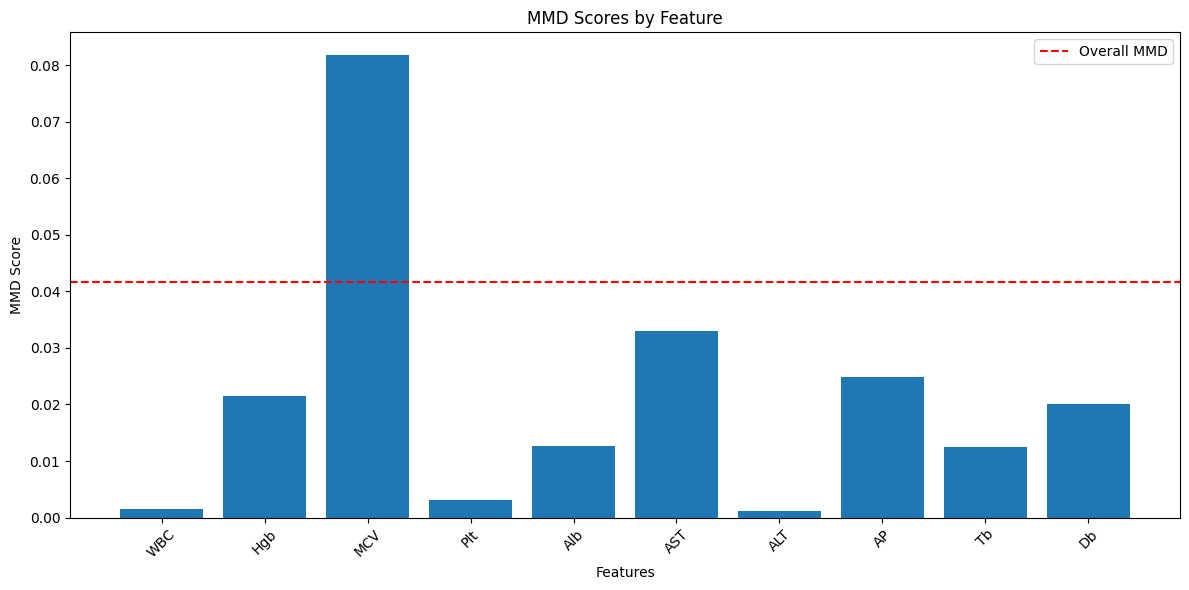
**

The figure shows the Maximum Mean Discrepancy (MMD) scores comparing real and synthetic data distributions for ten laboratory parameters (WBC: White Blood Cell count, Hgb: Hemoglobin, MCV: Mean Corpuscular Volume, Plt: Platelet count, Alb: Albumin, AST: Aspartate Aminotransferase, ALT: Alanine Aminotransferase, AP: Alkaline Phosphatase, Tb: Total Bilirubin, Db: Direct Bilirubin). The red dashed line indicates the overall MMD score of approximately 0.04 between the real and synthetic datasets. As noted in the manuscript, this low MMD score (close to 0) indicates that the distributions of the real and synthetic data are nearly identical. The individual bars represent feature-specific MMD scores, with MCV showing the highest discrepancy and most other parameters showing lower discrepancy values.

Supplementary Table 1

**Table 1: Performance Comparison of ML Algorithms Trained on Synthetic vs. Original Data, tested on real-world external validation set from MIMIC-III**

| **Algorithm** | **Synthetic Data Accuracy** | **Original Data Accuracy** | **Improvement** |
| --- | --- | --- | --- |
| KNN | 0.894 | 0.865 | +0.029 |
| LR | 0.900 | 0.882 | +0.018 |
| SVM | 0.935 | 0.865 | +0.070 |
| DT | 0.882 | 0.824 | +0.058 |
| NB | 0.800 | 0.659 | +0.141 |
| ANN | 0.924 | 0.882 | +0.042 |
| RF | 0.841 | 0.782 | +0.059 |

Abbreviations: KNN = K-Nearest Neighbors, LR = Logistic Regression, SVM = Support Vector Machine, DT = Decision Tree, NB = Naive Bayes, ANN = Artificial Neural Network, RF = Random Forest

Note: All algorithms were tested on the real-world external validation set from MIMIC-III. The difference in performance was statistically significant (p<0.01) across all algorithms.

**ICD-9 and ICD-10 Codes Used for Case Identification**

**Alcohol-associated Hepatitis**

- ICD-9: 571.1 (Acute alcoholic hepatitis)
- ICD-10: K70.10 (Alcoholic hepatitis without ascites)
- ICD-10: K70.11 (Alcoholic hepatitis with ascites)

**Acute Cholangitis**

- ICD-9: 576.1 (Cholangitis)
- ICD-10: K83.0 (Cholangitis)
